# Prediction models for phosphorus excretion of pigs

**DOI:** 10.5713/ab.24.0217

**Published:** 2024-08-16

**Authors:** Jeonghyeon Son, Beob Gyun Kim

**Affiliations:** 1 Department of Animal Science, Konkuk University, Seoul 05029, Korea

**Keywords:** Excretion, Phosphorus, Prediction Model, Swine

## Abstract

**Objective:**

The present study aimed to measure fecal and urinary phosphorus (P) excretion from pigs and to develop prediction models for P excretion of pigs.

**Methods:**

A total of 96 values for P excretions were obtained from pigs of 15 to 93 kg body weight (BW) fed 12 diets in four experiments and were used to develop the prediction models. All experimental diets contained exogenous phytase at 500 phytase units per kg. Body weight of pigs and dietary P concentrations were used as independent variables in the prediction models.

**Results:**

The BW, feed intake, and P intake were positively correlated with total (fecal plus urinary) P excretions (r = 0.80, 0.91, and 0.94, respectively; p<0.001). The models for estimating P excretion were: fecal P excretion (g/d) = −0.654–0.000618×BW^2^+0.273×BW ×dietary P concentration (R^2^ = 0.83; p<0.001); urinary P excretion (g/d) = 0.045+ 0.00781×BW×dietary P concentration (R^2^ = 0.15; p<0.001); total P excretion (g/d) = −0.598–0.000613×BW^2^+0.280×BW×dietary P concentration (R^2^ = 0.86; p<0.001) where the BW of pigs and dietary P concentration are expressed as kg and % (as-fed basis), respectively. Based on the developed prediction models, the estimated annual fecal, urinary, and total P excretion for a market pig was 1.24, 0.09, and 1.33 kg/yr, respectively.

**Conclusion:**

The P excretions in market pigs can be estimated using BW of pigs and dietary P concentration. In the present model, a market pig excretes 1.24 kg of fecal P and 0.09 kg of urinary P per year.

## INTRODUCTION

Phosphorus (P) is a major component of bones and plays a vital role in the maintenance and development of the skeletal system in pigs [1–3]. Therefore, an adequate supply of P is important for successful swine production [4,5]. However, undigested P is excreted in the feces, and a portion of digested P is excreted as urine. The excreted P is one of the main environmental pollutants derived from swine production [6]. An oversupply of P can increase the amount of P excretion, causing negative environmental impacts [7,8].

To efficiently manage the environmental pollution due to P excretion from pigs, an accurate measurement of P excretion is essential. In previous studies conducted in Europe [8,9] and the United States [10], the P excretion from pigs was estimated based on P intake and retained P. However, actual P output was not measured in these studies. In addition, when these studies were conducted in the 1990s, the use of phytase that reduces P excretion markedly was not yet widely used in swine diets. Therefore, the old estimations may not accurately represent the current P excretions from pigs fed diets mainly based on corn and soybean meal supplemented with phytase in the 2020s.

To our knowledge, fecal and urinary P excretion data for various growth stages of pigs are scarce. Moreover, a regression model for estimating P excretion from pigs based on recent data is unavailable. Therefore, the objectives of this study were to measure fecal and urinary P excretion from pigs and to develop the prediction equations for P excretion in market pigs using body weight (BW), feed intake (FI), and dietary P concentration with a hypothesis that P excretion is influenced by growth stage and dietary P concentration.

## MATERIALS AND METHODS

The protocols for animal experiments to obtain fecal and urinary samples were approved by the Institutional Animal Care and Use Committee of Konkuk University (Seoul, Republic of Korea; KU22046).

### Animals, diets, and sample collection

A total of 96 fecal and 96 urine samples were collected from pigs fed 12 diets in 4 experiments that were also used to determine nitrogen excretion from various stages of pigs [11,12]. All experiments were conducted using crossbred barrows (Landrace×Yorkshire) in an environmentally controlled room. Each of Exp. 1, 2, 3, and 4 was conducted using 12 barrows with initial BW of 15.2, 29.9, 50.2, and 78.2 kg, respectively (standard deviation = 0.7, 1.8, 2.2, and 3.7). In each experiment, a quadruplicated 3×2 incomplete Latin square design was employed with 3 diets of varying crude protein concentrations and 2 periods per square. At the end of the 2 periods, the final BW of each experiment was 24.7, 46.7, 70.0, and 92.6 kg, respectively (standard deviation = (1.4, 2.7, 4.2, and 4.5). The animals were individually housed in metabolism crates equipped with a feeder, a fully slatted floor, and a urine tray, facilitating the total, but separate collection of urine and feces from each pig. The mean P concentration in the experimental diets for Exp. 1, 2, 3, and 4 were 0.467%, 0.540%, 0.488%, and 0.428%, respectively (Table 1). All experimental diets were supplemented with phytase at 500 phytase units per kg. The standardized total tract digestible P concentrations for each ingredient were calculated by the exponential model suggested by Sung and Kim [1] to reflect the effect of phytase on P digestibility. Vitamins and minerals were included to meet or exceed the nutrient requirement estimates as suggested by the NRC [2].

In Exp. 1, the daily feed allowance was set at 6.0% of the initial BW to provide more than the amount of *ad libitum* FI and divided into three equal meals provided at 0800, 1230, and 1700 h. In Exp. 2, 3, and 4, the daily feed allowances were set at 4.5%, 4.0%, and 3.0% of the initial BW for each period, respectively, and divided into two equal meals served at 0800 and 1700 h. Pigs had free access to water. Each period consisted of a 5-day adaptation period followed by a 4-day collection period. On days 6 and 10, chromic oxide was added to the morning meals at a concentration of 0.5% as a marker, employing the marker-to-marker procedure for the quantitative feces collection [13]. Fecal collection started when the marker was first observed in the feces and concluded upon next appearance of the marker. Urine collection began at 1000 h on day 6 and ended at 1000 h on day 10. Buckets were emptied every morning and evening, and the collected urine was weighed, with a 10% subsample preserved at −20°C. Upon completing the sample collections, urine samples were thawed and mixed within each animal and diet.

### Chemical analyses

Fecal samples were dried in a forced-air drying oven at 55°C until the samples reached a constant weight. Diets and the dried fecal samples were finely ground for chemical analysis (<1 mm). Dry matter (2 h at 135°C; method 930.15), ash (method 942.05) [14], and gross energy (Parr 6200; Parr Instruments Co., Moline, IL, USA) in diets were determined. Diets were analyzed for nitrogen (method 990.03). Amylase-treated neutral detergent fiber (method 2002.04) concentrations in diet samples were analyzed with a heat-stable amylase and expressed inclusive of residual ash. Diets were analyzed for acid detergent fiber (method 973.18) [14]. Phosphorus in diets and fecal and urine samples was also analyzed (method 985.01) [14].

### Statistical analyses

An outlier in Exp. 2 was identified as the daily urinary P excretion deviated from the 1st or 3rd quartiles by more than three times the interquartile range. Correlation coefficients (r) among the BW, FI, dietary P concentrations, and daily P excretions were determined using the CORR procedure of SAS [15]. Prediction equations for daily fecal, urinary, and total P excretion were developed using the REG procedure of SAS [15]. Independent variables used to develop the equations were BW, FI, dietary P concentration, their squares, and their interactions. The model was selected using a stepwise selection procedure starting with guided forward selection through individual independent variables, with a p-value of less than 0.05 used to determine the inclusion of terms in the model. A pig was considered an experimental unit. The statistical significance level was declared at p<0.05.

### Calculations

The Gompertz model developed by Ahn et al [16] was used to estimate the daily BW of market pigs:


$$\text{BW}\;(\text{kg})=217.4e^{-4.6919e^{-0.0116t}}$$

where *t* represents the age of the market pigs in days (R^2^ = 0.999 and p<0.001). According to this prediction equation, the pigs reach a BW of 7 kg at 27 days of age and 121.5 kg at 180 days of age.

Daily fecal, urinary, and total P excretion (g/d) were calculated by substituting the BW (kg) estimated using the Gompertz model [16] and dietary P concentration (%) for each age into the developed prediction equations. The calculated daily P excretion for each day was averaged to estimate the growth stage-specific daily P excretion (g/d). The annual P excretion (kg/yr) was calculated by multiplying the daily P excretion (g/d) by 365 and then dividing the result by 1,000. The dietary P concentrations for pigs weighing 7 to 11, 11 to 25, 25 to 45, 45 to 65, 65 to 85, and 85 to 121.5 kg were 0.64%, 0.58%, 0.51%, 0.46%, 0.43%, and 0.40%, respectively. These concentrations align with the total P requirement estimates suggested by NIAS [17]. The dietary P concentrations are expressed as % on an as-fed basis. The BW ranges of 7 to 11, 11 to 25, 25 to 45, 45 to 65, 65 to 85, and 85 to 121.5 kg correspond to the age ranges of 27 to 39, 40 to 66, 67 to 94, 95 to 117, 118 to 138, and 139 to 180 days, respectively.

## RESULTS

For 15-kg pigs, the average daily fecal, urinary, and total P excretion was 1.54, 0.09, and 1.63 g/d, respectively (Table 2). The average daily fecal, urinary, and total P excretion for 30-kg pigs was 3.27, 0.26, and 3.53 g/d, respectively. For 50-kg pigs, the average daily fecal, urinary, and total P excretion was 5.02, 0.16, and 5.18 g/d, respectively. The average daily fecal, urinary, and total P excretion for 80-kg pigs was 4.62, 0.35, and 4.97 g/d, respectively.

The BW, FI, and P intake were positively correlated with fecal, urinary, and total P excretions (p<0.01; Table 3). Best-fitting prediction equations for the daily P excretion were developed (Table 4). Equations 1 to 3 are based on BW and dietary P concentration and Eq. 4 to 6 are based on FI, dietary P concentration, or both.

**Figure 1:**
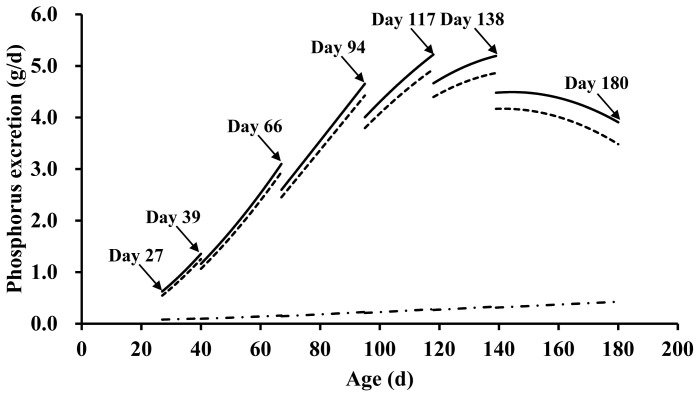
Estimated fecal (dotted line), urinary (dash-dotted line), and total (solid line) phosphorus (P) excretion (g/d) of pigs for each age. Pigs aged 27 to 39 (phase 1), 40 to 66 (phase 2), 67 to 94 (phase 3), 95 to 117 (phase 4), 118 to 138 (phase 5), and 139 to 180 days (phase 6) weighed 7 to 11, 11 to 25, 25 to 45, 45 to 65, 65 to 85, and 85 to 121.5 kg, respectively. The dietary P concentration for phases 1, 2, 3, 4, 5, and 6 was set at 0.64, 0.58, 0.51, 0.46, 0.43, and 0.40% on an as-fed basis, respectively. Average daily fecal P excretion for phases 1, 2, 3, 4, 5, and 6 was 0.85, 1.91, 3.40, 4.37, 4.66, and 3.95 g/d, respectively. Average daily urinary P excretion for phases 1, 2, 3, 4, 5, and 6 was 0.09, 0.12, 0.18, 0.24, 0.30, and 0.37 g/d, respectively. Average total P excretion for phases 1, 2, 3, 4, 5, and 6 was 0.95, 2.04, 3.58, 4.62, 4.96, and 4.32 g/d, respectively. The average daily fecal, urinary, and total P excretion for pigs at 27 to180 days of age was 3.39, 0.24, ad 3.64 g/d, respectively. The estimated annual fecal, urinary, and total P excretion for pigs was 1.24, 0.09, and 1.33 kg/yr, respectively. The body weight (BW) of pigs was estimated using the Gompertz model developed by Ahn et al [16]. Dietary P concentrations met the total P requirement estimates suggested by the NIAS [17]. Daily fecal and urinary P excretion values were obtained based on following equations: fecal P excretion = −0.654–0.000618×BW^2^ (kg)+0.273×BW×diet P (%, as-fed basis); urinary P excretion = 0.045+0.00781×BW×diet P. Total P excretion values were calculated by summing fecal P excretion and urinary P excretion.

Based on the equations for fecal and urinary P excretion (Eq. 1 and 2) using BW and dietary P concentration, the average daily fecal, urinary, and total P excretion for pigs from 7 to 121.5 kg BW at 27 to 180 days of age was 3.39, 0.24, and 3.64 g/d, respectively (Figure 1). The estimated annual fecal, urinary, and total P excretion for pigs were 1.24, 0.09, and 1.33 kg/yr, respectively.

## DISCUSSION

Phosphorus excreted from pig production has been claimed to be a source of environmental pollution [7,8]. An accurate estimation of P excretion from pigs is critical for making policies for environmental management. However, previous models from Europe [8,9] and the United States [10] for estimating P excretion from pigs were based on data mostly in 1990s and did not likely consider the use of exogenous phytase in the diets. Novel models for estimating P excretion from pigs fed corn-soybean meal-based diets supplemented with phytase were developed in the present work. The consideration of exogenous phytase is important for estimating P excretion from pigs because current swine diets are mostly added with phytase to increase P digestibility of plant ingredients [1,18,19]. In the present study, all experimental diets were supplemented with phytase at 500 phytase units per kg of diet to represent the current commercial swine diets.

In the present study, the total P excretion quadratically increased as the BW of pigs increased, which is likely due to the quadratic increase in P intake as the BW of pigs increased. The requirement of P as gram per day increase in a quadratic manner with pig BW. Although the dietary P concentration decreases as pigs grow, the quadratic increase of FI with pig BW apparently overrides the changes of dietary P concentrations.

The fecal P excretion is the primary contributor to the total P excretion of pigs [6,20]. This is because the feed ingredients used in this study are primarily plant-based ingredients containing high concentrations of phytate-P which is not very digestible in pigs [21]. Thus, a substantial amount of ingested P is excreted as feces. In addition, a large portion of digested and absorbed P is retained in growing pigs, resulting in a relatively small proportion of urinary P excretion. Based on these observations, reducing fecal P excretion would be an effective strategy to decrease environmental pollution due to P excretion from pig production.

Using the correlation analysis, candidate independent variables for estimating P excretions were screened. Based on the strong correlation between BW and P excretion, BW was selected as a candidate of independent variable for the equations. As a pig’s BW increases, the quantity of daily P intake increases, and thus, fecal P output would also increase. With the same token, FI was also selected as a candidate independent variable for the model. Although P intake itself could also be a strong candidate for explaining P excretion, P intake data are not easily obtainable. Alternatively, BW×diet P which partially represents P intake was used as an explanatory variable for P excretion.

The daily fecal and urinary P excretion was calculated using Eq. 1 and 2, respectively, and then the mean daily P excretion from pigs of 27 to 180 day was multiplied by 365 to obtain the annual P excretion from pigs weighing 7 to 121.5 kg BW. The estimated annual total (fecal plus urinary) P excretion for market pigs was 1.33 kg/yr in the present study. This estimate is less than the value for France, Denmark, and the Netherlands (2.5, 2.3, and 1.7 kg/yr, respectively) reported by Jongbloed et al [9] in 1999. Our estimate is also less than the previous values for annual total P excretion (2.57 and 2.09 kg/yr) of market pigs estimated by Dourmad et al [8] in 1999 and by Carter et al [10] in 2003. Apparently, pig diet formulations have dramatically changed during the past two decades. Particularly dietary P concentrations of pig diets in these days are much less than the diets used 25 years ago mainly due to the increased use of exogenous phytase [22]. The reduced dietary P concentrations are likely to lower the quantity of P excretion and supplemental phytase additionally lower fecal P excretion by increasing P digestibility [1,19].

In the present estimation of annual P output from pig production, the exogenous phytase concentration was assumed to be 500 phytase units per kg of diet. A higher dose of phytase would even lower P excretion from pigs by increasing P digestibility and lowering dietary P concentrations during diet formulations. In addition, the experimental diets used for modeling the equations were based mainly on corn and soybean meal in the present study. If the ingredient composition largely changes, the amount of P excretion may also change. The growth curve [16] and FI [2] used in the present work are also important assumptions. The marketing age of pigs was assumed to be 180 days (121.5 kg BW) in this work. If the marketing age is delayed to reach the slaughter weight, the annual P excretion may increase.

A limitation of this work is that two-way crossbred pigs were used in the animal experiments. Although the breed of pigs used in this study differs from the three-way crossbred or hybrid pigs widely reared in commercial farms, the effects of genotype on the P excretion have been reported to be negligible [23,24]. Additionally, although only barrows were used in the present experiments, the P digestibility is not affected by the sex [25] and the growth model used to estimate P excretion was developed based on the growth model for barrows and gilts at 1:1 [2].

Another limitation of this study is that the P excretions from gestating and lactating sows were not considered. Based on the assumption that sows excrete approximately 2 times the amount of P compared with marketing pigs and the population of sows is approximately 10% of total pig population, the annual P excretion from a pig would be 1.46 kg/yr.

In conclusion, the excretions of phosphorus in market pigs can be estimated using body weight of pigs and the dietary phosphorus concentrations. Based on the prediction models developed in the present study, the annual fecal, urinary, and total phosphorus excretion was 1.24, 0.09, and 1.33 kg/yr for pigs from 7 to 121.5 kg body weight, respectively.

## Figures and Tables

**Table 1:** Ingredient and chemical composition of experimental diets^1)^ (as-fed basis)

Items	BW: 15 to 25 kg (Exp. 1)			30 to 47 kg (Exp. 2)			50 to 70 kg (Exp. 3)			78 to 93 kg (Exp. 4)		
			
	CP: 19.4%	17.5%	15.7%	16.8%	15.0%	13.2%	16.8%	14.9%	13.1%	15.1%	13.2%	11.4%
Ingredient (%)												
Ground corn	65.12	71.01	76.72	68.50	73.93	79.68	69.16	74.68	79.97	78.19	83.62	88.99
SBM (45% CP)	17.60	10.90	4.20	15.50	9.50	2.80	16.00	10.10	4.20	18.70	12.90	7.00
Whey powder	8.00	8.00	8.00	-	-	-	-	-	-	-	-	-
Fish meal	4.00	4.00	4.00	-	-	-	-	-	-	-	-	-
SDPP	2.00	2.00	2.00	-	-	-	-	-	-	-	-	-
Corn DDGS	-	-	-	8.00	8.00	8.00	5.50	5.50	5.50	-	-	-
Rapeseed meal	-	-	-	4.00	4.00	4.00	6.00	6.00	6.00	-	-	-
L-Lys·HCl (78.8%)	0.38	0.59	0.80	0.43	0.62	0.83	0.23	0.41	0.59	0.12	0.30	0.48
DL-Met (99.0%)	0.11	0.20	0.30	0.10	0.18	0.28	0.01	0.10	0.18	-	0.08	0.16
L-Thr (98.0%)	0.11	0.17	0.24	0.05	0.10	0.17	-	0.02	0.07	-	0.02	0.07
L-Trp (98.0%)	-	0.04	0.08	0.02	0.05	0.09	-	0.03	0.06	-	0.01	0.05
L-Ile (98.5%)	-	0.08	0.20	-	0.05	0.17	-	-	0.08	-	-	0.05
L-Val (98.0%)	-	0.12	0.23	-	0.09	0.21	-	-	0.09	-	-	0.07
L-Phe (98.0%)	-	0.08	0.20	-	0.01	0.14	-	-	0.03	-	-	-
L-His (98.5%)	-	0.05	0.11	-	-	0.07	-	-	-	-	-	-
L-Leu (99.0%)	-	-	0.09	-	-	-	-	-	-	-	-	-
Soybean oil	1.00	1.00	1.00	1.00	1.00	1.00	1.00	1.00	1.00	1.00	1.00	1.00
Sodium chloride	0.20	0.20	0.20	0.30	0.30	0.30	0.30	0.30	0.30	0.30	0.30	0.30
Ground limestone	1.06	1.05	1.03	1.00	1.00	1.00	0.97	0.99	0.98	0.86	0.88	0.87
Dicalcium phosphate	0.11	0.20	0.29	0.79	0.86	0.95	0.52	0.56	0.64	0.52	0.58	0.65
Phytase^2)^	0.01	0.01	0.01	0.01	0.01	0.01	0.01	0.01	0.01	0.01	0.01	0.01
Vitamin-mineral premix^3)^	0.30	0.30	0.30	0.30	0.30	0.30	0.30	0.30	0.30	0.30	0.30	0.30
Analyzed composition												
Gross energy (kcal/kg)	3,935	3,944	3,941	4,001	4,002	3,930	3,939	3,950	3,893	3,906	3,871	3,863
Dry matter (%)	88.8	88.3	88.6	88.9	89.1	87.8	87.4	87.2	87.0	87.0	86.9	86.7
Ash (%)	4.61	4.17	4.03	4.73	4.49	4.14	4.46	4.21	4.03	4.09	3.71	3.51
CP (%)	18.8	17.5	15.6	17.6	15.6	14.2	15.5	13.2	10.2	14.1	12.7	11.1
aNDF (%)	8.16	8.33	7.58	11.66	11.53	11.51	11.74	11.63	11.93	8.85	9.13	8.80
ADF (%)	2.58	1.72	2.28	3.58	3.46	3.06	4.66	4.44	4.20	2.90	2.67	2.55
Phosphorus (%)	0.445	0.495	0.460	0.555	0.540	0.525	0.500	0.480	0.485	0.450	0.420	0.415

BW, body weight; CP, crude protein; SBM, soybean meal; SDPP, spray-dried plasma protein; DDGS, distillers dried grain with solubles; aNDF, amylase-treated neutral detergent fiber; ADF, acid detergent fiber.

1) The ingredient and chemical composition of experimental diets were derived from the previous experiments that aimed to determine nitrogen balance of pigs [11,12].

2) The phytase products (Natuphos® E 5000 G; BASF Corporation, Florham Park, NJ, USA) contained approximately 5,000 phytase unit (FTU)/g of phytase activity, and the experimental diets contained phytase to provide 500 FTU per kg of diet.

3) The vitamin-mineral premix provided the following quantities per kilogram of complete diet: vitamin A, 24,000 IU; vitamin D_3_, 3,600 IU; vitamin E, 120 mg; vitamin K, 3.0 mg; thiamin, 6.0 mg; riboflavin, 9.0 mg; pyridoxine, 4.2 mg; vitamin B_12_, 0.036 mg; pantothenic acid, 27.0 mg; folic acid, 0.615 mg; niacin, 33.0 mg; biotin, 0.6 mg; Cu, 13.2 mg as copper sulfate; Fe, 75.36 mg as iron sulfate; I, 1.16 mg as calcium iodate; Mn, 12.84 mg as manganese sulfate; Zn, 35.7 mg as zinc sulfate; Co, 0.006 mg as cobaltous carbonate; and Se, 0.20 mg as selenomethionine.

**Table 2:** Range and variability of body weight, feed intake, and phosphorus (P) utilization of pigs^1)^

Items	N	Mean	Minimum	Maximum	SD	CV
Exp. 1 (15 to 25 kg)						
Initial body weight (kg)	24	17.6	13.8	22.8	2.7	15.1
Final body weight (kg)	24	22.3	18.1	27.9	2.8	12.5
Feed intake (g/d)	24	924	694	1,268	164	17.8
P intake (g/d)	24	4.31	3.39	5.64	0.77	17.9
Fecal P excretion (g/d)	24	1.54	0.75	2.48	0.43	27.9
Urinary P excretion (g/d)	24	0.09	0.01	0.41	0.11	120.3
Total P excretion (g/d)	24	1.63	0.84	2.51	0.45	27.6
Exp. 2 (30 to 47 kg)						
Initial body weight (kg)	23	33.3	27.8	40.9	4.2	12.6
Final body weight (kg)	23	42.3	33.5	51.1	5.5	12.9
Feed intake (g/d)	23	1,477	1,229	1,841	204	13.8
P intake (g/d)	23	7.97	6.52	10.21	1.12	14.0
Fecal P excretion (g/d)	23	3.27	1.80	5.11	0.84	25.7
Urinary P excretion (g/d)	23	0.26	0.02	0.98	0.29	111.2
Total P excretion (g/d)	23	3.53	2.07	5.22	0.86	24.4
Exp. 3 (50 to 70 kg)						
Initial body weight (kg)	24	54.2	45.3	62.2	4.8	8.8
Final body weight (kg)	24	64.1	53.0	76.7	6.9	10.7
Feed intake (g/d)	24	2,169	1,812	2,488	190	8.7
P intake (g/d)	24	10.59	9.06	12.44	0.95	8.9
Fecal P excretion (g/d)	24	5.02	3.33	7.08	1.09	21.7
Urinary P excretion (g/d)	24	0.16	0.01	0.42	0.13	77.3
Total P excretion (g/d)	24	5.18	3.41	7.12	1.07	20.6
Exp. 4 (78 to 93 kg)						
Initial body weight (kg)	24	81.8	71.1	89.3	5.4	6.6
Final body weight (kg)	24	89.0	76.2	96.7	5.7	6.4
Feed intake (g/d)	24	2,453	2,133	2,679	163	6.6
P intake (g/d)	24	10.51	8.91	12.06	0.81	7.7
Fecal P excretion (g/d)	24	4.62	3.64	5.79	0.59	12.8
Urinary P excretion (g/d)	24	0.35	0.16	0.72	0.17	47.6
Total P excretion (g/d)	24	4.97	3.84	6.25	0.60	12.1

SD, standard deviation; CV, coefficient of variation.

1) Initial body weight, final body weight, and feed intake data were obtained from the previous experiments [11,12]. The body weight and feed intake values are the means of periods 1 and 2.

**Table 3:** Correlation coefficients among body weight, feed intake, dietary phosphorus (P) concentration, and P utilization of pigs^1)^

Items	Body weight	Feed intake	Dietary P concentration	P intake	Fecal P excretion	Urinary P excretion
Feed intake	0.96^***^					
Dietary P concentration	−0.51^***^	−0.36^***^				
P intake	0.87^***^	0.96^***^	−0.10			
Fecal P excretion	0.78^***^	0.90^***^	−0.12	0.93^***^		
Urinary P excretion	0.38^***^	0.33^**^	0.05	0.32^**^	0.20	
Total P excretion	0.80^***^	0.91^***^	−0.12	0.94^***^	0.99^***^	0.32^**^

1) A pig was considered as an experimental unit (n = 95).

** p<0.01 and

*** p<0.001.

**Table 4:** Prediction equations for daily phosphorus (P) excretion (g/d) of pigs^1)^

Items	Equation^2)^	Statistical parameter		

		RMSE	R^2^	p-value
Eq. 1	Fecal P excretion = −0.654–0.000618×BW^2^+0.273×BW×diet P	0.656	0.83	<0.001
Eq. 2	Urinary P excretion = 0.045+0.00781×BW×diet P	0.194	0.15	<0.001
Eq. 3	Total P excretion = −0.598–0.000613×BW^2^+0.280×BW×diet P	0.624	0.86	<0.001
Eq. 4	Fecal P excretion = −0.855+5.35×FI×diet P	0.572	0.87	<0.001
Eq. 5	Urinary P excretion = 0.023+0.11×FI	0.198	0.11	0.001
Eq. 6	Total P excretion = −0.842+5.60×FI×diet P	0.550	0.89	<0.001

RMSE, root mean square of error; R^2^, coefficient of determination; BW, body weight (kg); diet P, total phosphorus concentration of diet (% as-fed); FI, feed intake (kg/d).

1) A pig was considered as an experimental unit (n = 95).

2) All independent variables and the intercept were significant, excluding the intercepts in Eq. 2 and Eq. 5.
